# Histone methyltransferase EZH2 drives podocyte injury and senescence in diabetic nephropathy through STAT3 activation

**DOI:** 10.1007/s00018-026-06136-x

**Published:** 2026-03-11

**Authors:** Mengfei He, Lulu Liang, Panpan Zhou, Linxiao Lv, Mingyang Hu, Shaokang Pan, Mianzhi Zhang, Zhangsuo Liu, Dongwei Liu, Sijie Zhou

**Affiliations:** 1 Department of Nephrology, the First Affiliated Hospital of Zhengzhou University, Zhengzhou, 450052 P. R. China; 2 Research Institute of Nephrology, Zhengzhou University, Zhengzhou, 450052 P. R. China; 3 Henan Province Research Center For Kidney Disease, Zhengzhou, 450052 P. R. China; 4 Key Laboratory of Precision Diagnosis and Treatment for Chronic Kidney Disease in Henan Province, Zhengzhou, 450052 P. R. China; 5 Dongfang Hospital of Beijing University of Chinese Medicine, Beijing, 100078 China; 6 Tianjin Academy of Traditional Chinese Medicine, Tianjin, 300120 China

**Keywords:** Diabetic nephropathy, Enhancer of zeste homolog 2, Signal transducer and activator of transcription 3, Podocyte injury, Cellular senescence

## Abstract

**Background:**

As a common secondary nephropathy, diabetic nephropathy (DN) is closely related to podocyte senescence. Histone methyltransferase enhancer of zeste homolog 2 (EZH2) participates in the regulation of cellular proliferation, apoptosis, and senescence. Recently, the non-histone role of EZH2 has attracted much attention. It was reported that EZH2 can directly combine with signal transducer and activator of transcription 3 (STAT3) thereby enhancing its activity. However, the association between EZH2 and STAT3 in podocyte senescence remains unclear.

**Methods:**

To clarify the association between EZH2 and STAT3 and their functions in podocyte injury and senescence in DN, we established db/db mice and cultured mouse podocyte cells (MPCs) exposed to high glucose (HG) as DN models. EZH2 was regulated genetically and pharmacologically in this study and changes in indicators related to podocyte injury and aging were detected.

**Key findings:**

Elevated levels of EZH2, inflammatory markers, and reduced podocyte marker proteins were observed in db/db mice and HG-cultured MPCs. Additionally, aggravated podocyte senescence was observed in the DN group. Pharmacological inhibition of EZH2 by GSK126 alleviated kidney aging and podocyte injury markers by approximately 54% in db/db mice. Moreover, we confirmed that EZH2 can bind to STAT3 and increase the methylation of its lysine residues, thereby promoting its activity. However, rescue experiments in vivo and in vitro revealed that the beneficial effect of EZH2 downregulation was counteracted by overexpression of STAT3.

**Conclusion:**

Mechanistically, EZH2 interacted with STAT3 and enhanced its activation, in association with increased lysine methylation of STAT3. Together, our findings identify EZH2-STAT3 signaling as a key driver of podocyte injury and senescence in DN and suggest that targeting EZH2 may represent a promising therapeutic strategy for DN.

**Supplementary Information:**

The online version contains supplementary material available at 10.1007/s00018-026-06136-x.

## Introduction

Diabetes mellitus (DM) is a significant global threat to human health. Statistics from the International Diabetes Federation suggest that, globally, the number of patients has increased to 537 million in 2021 [1]. Diabetic nephropathy (DN), a common complication in patients with long-term DM with symptoms of increased urinary albumin excretion and impaired renal function, is gradually becoming the primary cause of chronic kidney disease, which eventually leads to end-stage renal disease [2–4]. Typical alterations involving renal structures in DN include the thickening of glomerular basement membrane (GBM), podocyte detachment and foot process effacement, mesangial matrix expansion, and endothelial fenestration loss [5]. Podocytes are terminally differentiated epithelial cells and an indispensable component of the glomerular filtration barrier. According to extensive research, podocytes are highly associated with the occurrence of proteinuria in DN patients [6].

Cell senescence is the reaction that occurs in response to various forms of cellular stress, characterized by permanent growth arrest and altered secretory phenotype. Despite senescent cells have withdrawn from normal cell cycle, they still sustain their metabolic function, releasing cytokines and chemokines, and promoting the neighboring cells to enter the inflammatory state through paracrine signaling, which can promote glomerulosclerosis in DN [7]. Integrated Bioinformatics Analysis showed that 12 hub senescence-related genes in glomerular cells exhibited a significantly higher senescence score than other kidney cell clusters in the early stage of DN mice [8]. Notably, studies have indicated that podocyte senescence is essential in various pathogenic renal processes [9]. This role likely involves regulating oxidative stress, mitochondrial dysfunction, pyroptosis, and apoptosis [10].

Histone methyltransferase enhancer of zeste homolog 2 (EZH2), a catalytic component of polycomb repressive complex 2 (PRC2), represses gene transcription by catalyzing histone H3 lysine 27 trimethylation (H3K27me3) [11, 12]. Studies have shown that EZH2 is involved in the regulation of cellular senescence in atrial fibrosis, acute kidney injury, irradiation-induced kidneys, liver fibrosis, gestational system diseases and cancers [13–15]. In addition to histone modification, the non-histone role of EZH2 has also attracted attention. It was reported that EZH2 can methylate non-histone substrates, especially transcription factors and chromatin—related proteins, such as GATA binding protein 4, signal transducer and activator of transcription 3 (STAT3) and androgen receptors [16–19]. Of note, STAT3 is reported to participate in the processes of cellular senescence, inflammation, renal fibrosis and extracellular matrix accumulation in DN. However, the non-canonical role of EZH2 and the relationship between EZH2 and STAT3 in DN remain unclear.

To explore the effects of EZH2 on DN, we determined the expression of EZH2 in DN models in vivo and in vitro. EZH2 inhibitor (GSK126), adenoviruses, and podocyte-specific adeno-associated viruses were used to investigate the roles and mechanisms involving EZH2 in podocytes in DN. We found that EZH2 physically interacted with STAT3, enhanced its phosphorylation and transcriptional activity, and was associated with increased lysine methylation on STAT3 in diabetic conditions.

## Materials and methods

### Clinical studies

All renal biopsy specimens were acquired from the First Affiliated Hospital of Zhengzhou University. The diagnosis of DN was established histopathologically, and cases with coexisting other kidney diseases or a history of treatment with glucocorticoids, immunosuppressants and cytotoxic drugs were excluded. Paraffin-embedded kidney sections of DN patients (*n* = 10) were collected from the renal pathology laboratory. Control group (*n* = 10) kidney tissues were collected from patients suffering from surgical full or partial nephrectomy without primary or secondary kidney disease and the renal dysfunction. This investigation was approved by the Institutional Ethics Committee of the First Affiliated Hospital of Zhengzhou University (Ethics Approval No. 2023-KY-0656-002).

### Establishment of animal models

All animal studies were performed in accordance with the National Institutes of Health guidelines for animal experiments and under the review and approval of the Ethics Committee of the Experimental Animal Center of Zhengzhou University (Ethics Approval No. ZZU-LAC20220722 [19]). Due to the spontaneous development of type 2 DM and symptoms of DN, such as microproteinuria, typically observed at 10 weeks in db/db mice, we chose db/db mice as an in vivo DN model with db/m mice as control. All animals used in this study were purchased from Gempharmatech Co., Ltd. (Jiangsu, China). Male db/db mice aged 8-weeks were acclimated for 2 weeks prior to experimentation.

At the age of 10-weeks, the male db/m and db/db mice were fed without any intervention until 22 weeks and then euthanized before specimen collection (*n* = 6).

Male db/db mice, also aged 10 weeks, were allocated into the following groups at random: (1) db/db group (*n* = 6): untreated mice; (2) db/db + Sulfobutylether-β-Cyclodextrin (SBE-β-CD) group (*n* = 6): mice were continuously injected solvent SBE-β-CD intraperitoneally for 5-days per week over 8-weeks; (3) db/db mice + GSK126 group (*n* = 6): mice were continuously injected with EZH2 inhibitor GSK126 (50 mg/kg) intraperitoneally for 5-days per week over 8-weeks. All mice were euthanized, and their kidneys were collected for analysis at 18-weeks of age. SBE-β-CD (HY-17031) and GSK126 (HY-13470) were purchased from MedChemExpress (Shanghai, China).

At 10-weeks of age, male db/db mice were randomly divided into different cohorts as follows: (1) db/db group (*n* = 6): untreated mice; (2) db/db + vehicle group (*n* = 6): mice were injected with an adeno-associated virus vector (AAV) carrying the podocyte-specific promoter via tail-vein at the ages of 10- and 16-weeks; (3) db/db + EZH2 KD group (*n* = 6): mice were injected with an AAV carrying the podocyte-specific EZH2 small hairpin RNA via the tail-vein at the ages of 10- and 16-weeks; (4) db/db + EZH2 KD + STAT3 OE group (*n* = 6): mice were injected with an AAV containing an EZH2 knockdown sequence and an AAV designed to induce STAT3 overexpression via tail-vein at the ages of 10- and 16-weeks. To maximize the effects of the AAVs, the mice were euthanized before their kidneys were collected at 22 weeks of age.

Fasting blood glucose levels and urinary albumin/creatinine ratios (uACR) of all mice were monitored. Each isolated kidney was dissected into suitable sizes and stored under appropriate conditions for subsequent experiments.

### Cell culture and transfection

The conditionally cultured immortalized mouse podocyte cells (MPCs) used in our study were kindly donated by the Jinling Clinical Medical College of Nanjing Medical University. MPCs were proliferated at 33℃ and differentiated at 37℃ in RPMI1640 medium (Gibco, New York, USA) supplemented with glucose, 1% penicillin-streptomycin, and 10% fetal bovine serum (FBS, Gibco, New York, USA). At 37℃, the differentiated MPCs were stimulated with medium containing normal glucose, high mannitol, high glucose, stattic (HY-13818, MedChemExpress, Shanghai, China), and adenoviruses, respectively. All adenoviruses used to knock down EZH2 or overexpress STAT3 and control empty carriers employed in this study were established and provided by Hanbio Biotechnology Co., Ltd. (Shanghai, China).

The MPCs were randomly allocated into the following groups once the cells had differentiated and matured at 37℃: (1) normal glucose (NG) group: MPCs were cultured in medium with a glucose concentration of 5 mM; (2) high mannitol (HM) group: MPCs were stimulated in high mannitol (20 mM) medium; and (3) high glucose (HG) group: MPCs were stimulated with high glucose (33.3 mM) medium to simulate a diabetic environment.

Differentiated and matured MPCs were randomly divided into different groups: (1) HG group: MPCs were treated with HG medium as a control group; (2) HG + vehicle group: MPCs were treated with empty carrier adenovirus under HG conditions; (3) HG + EZH2 knockdown (KD) group: MPCs were transfected with EZH2 KD adenovirus under HG conditions; and (4) HG + EZH2 KD + STAT3 overexpression (OE) group: MPCs were transfected with both EZH2 KD and the STAT3 OE adenovirus under HG conditions.

MPCs were divided into three groups as following: (1) HG group: MPCs were treated with HG medium as a control group; (2) HG + EZH2 KD group; (3) HG + EZH2 KD + stattic group: MPCs were transfected with EZH2 KD adenovirus and treated with stattic (5µM) under HG environment.

All adenoviruses were utilized in accordance with the protocols specified by the manufacturer. Before transfection, the viruses were allowed to thaw slowly on ice. The required amount of virus was added to half a volume of the needed culture medium and mixed thoroughly. The mixture was then added to the cell culture flasks, and the remaining half of the culture medium was added after 4-h incubation at 37℃. The next morning, the medium containing viruses was replaced by fresh culture medium according to the experimental groups involved. Finally, cells were collected for further experiments 36-h later.

### Isolation of glomeruli

The method to isolate glomeruli from mice is described in our previous studies [20, 21]. Briefly, the collected kidneys were removed after perfusion and cut into 1mm^3^ pieces and digested in collagenase A at 37 °C. After grinding, the kidney tissue was filtered through a 100 μm cell sieve and the glomeruli were collected.

### Western blotting

Glomeruli isolated from the kidneys of db/db mice and cultured podocytes were added to pre-cooled radioimmunoprecipitation assay lysis buffer with protease inhibitors (CW2200S, CWBio, Beijing, China), phosphatase inhibitors (CW2383S, CWBio, Beijing, China), and PMSF (P0100, Solarbio, Beijing, China) and incubated for > 30 min on ice. Then, the protein lysate was centrifuged at 12,000 rpm at 4℃ for 10 min to obtain supernatants. Next, 5× SDS loading buffer was added to the supernatant and boiled for 10 min at 99℃. Subsequently, the protein samples were analyzed using western blotting. All antibodies used in this study were purchased from commercial vendors and are listed in Table S1. Secondary antibodies were acquired from Abbkine Scientific Co. Ltd. (Wuhan, China).

### Co-immunoprecipitation (Co-IP)

Protein lysates were prepared using Pierce™ IP lysate buffer (Thermo Fisher Scientific, Shanghai, China), protease inhibitors, phosphatase inhibitors, and PMSF and added to culture flasks washed with phosphate buffered saline (PBS). Cells were scraped and centrifuged using the same protocol employed to extract protein supernatants. Dynabeads™ protein G beads (Invitrogen, USA) were washed in IP lysate buffer and then added to the protein supernatant. The mixture was incubated on a rotating rack for 1-h, after which the supernatant was collected. Part of the supernatant was used as the Input group and boiled for denaturation in 5× SDS loading buffer. The remaining cell lysis buffer was equally divided into IP and IgG groups, and EZH2 (or STAT3) and IgG antibodies were added to these two groups. After mixing at 4℃ for 2-h, equal amounts of cleaned beads and appropriate amount of PMSF (PMSF: lysate = 1:100) were added for overnight incubation. On day 2, the supernatant was discarded, and the beads were washed and denatured by boiling in 1× loading buffer. The supernatant was then retained for western blotting analysis.

### Quantitative real-time PCR

Total RNA was extracted from cultured MPCs using RNeasy Mini Kit (Qiagen, Hilden, Germany) according to the manufacturer’s instructions. The concentration and purity of RNA were detected using an Eppendorf Biospectrometer spectrophotometer. RNA was subsequently diluted into 300 ng/µl in RNAse-free water. Then, complementary DNA was synthesized by reverse transcription using RevertAid First Strand cDNA Synthesis Kit (Thermo Fisher Scientific, Shanghai, China). Oligo (dT) primer was used for *Ezh2* reverse transcription according to the manufacturer’s instructions. Quantitative real-time PCR (qPCR) was employed to measure relative expression levels of target genes using Maxima SYBR Green qPCR Master Mix (Thermo Fisher Scientific, Shanghai, China). The primers were designed and synthesized by Sangon Biotech (Shanghai, China) (Table S2).

### Immunofluorescence staining

Cultured podocytes and frozen tissue sections were fixed in 4% paraformaldehyde for 30 min. The cells and sections were permeabilized with 0.1% Tiriton-100 for approximately 30 min and blocked with 3% bovine serum albumin (BSA) for 20 min. Next, the samples were incubated at 4 °C overnight with primary antibodies diluted in 1% BSA. The following antibodies were used for immunofluorescence staining: EZH2 (1:100, 5246 S, CST, USA), pSTAT3 (1:50, 4113 S, CST, USA), podocin (1:200, ab50339, Abcam, UK), WT-1 (1:200, ab89901, Abcam, UK), Flag (1:200, 80010-1-RR, Proteintech, Wuhan, China) and B7-1 (1:200, sc-376012, Santa Cruz Biotechnology, USA). Finally, images were captured and analyzed using a Zeiss LSM880 confocal microscope (Carl Zeiss, Germany).

### Immunohistochemistry staining

Formalin-fixed, paraffin-embedded renal biopsy tissues of DN patients and mouse kidney tissues were cut into 4 μm-thick sections. A periodic acid-Schiff (PAS) staining kit (1.01646, Sigma-Aldrich, USA) was used for PAS staining according to the manufacturer’s protocol. Immunohistochemistry staining was performed using primary antibodies against p16 (1:200, sc-1661, Santa Cruz Biotechnology, USA) and p21 (1:200, ab188224, Abcam, UK). Nucleus staining was performed with hematoxylin dye, followed by mounting with neutral gum. Images were captured using an Olympus BX53F microscope (Tokyo, Japan).

### Senescence-associated β-galactosidase (SA-β-gal) staining

Accumulation of β-galactosidase is a characteristic of senescent cells. A specific staining kit was obtained from Beyotime Biotechnology (C0602, Shanghai, China) for the detection of SA-β-gal. Experiments were performed in accordance with the manufacturer’s instructions. Nucleus Fast Red (G1320, Solarbio Science and Technology Co., Ltd, Beijing, China) was employed to counterstain the frozen tissue sections.

### Transmission electron microscopy (TEM)

Renal cortex tissues were cut into 1-mm^3^ pieces, immediately immersed in precooled 4% glutaraldehyde solution for prefixation, and then post-fixed in 1% osmic acid. After washing with PBS, samples were subjected to gradient dehydration with acetone and embedded in Epon 812 epoxy resin (Canemco 034). Transmission electron micrographs were obtained using a JEOL JEM-1400 Plus transmission electron microscope (JEOL Inc., Peabody, MA, USA) at 80 kV.

### Statistical analysis

Six replicates were used for each group in in vivo studies, and three replicates were used for in vitro studies. Data with normal distributions were analyzed using Student’s *t*-test. One-way ANOVA was performed for comparisons between two or more groups using GraphPad Prism. Statistics are expressed as mean ± standard deviation. Statistical significance was set at *P* < 0.05.

## Results

### EZH2 upregulation and aggravated renal damage in DN

**Fig. 1:**
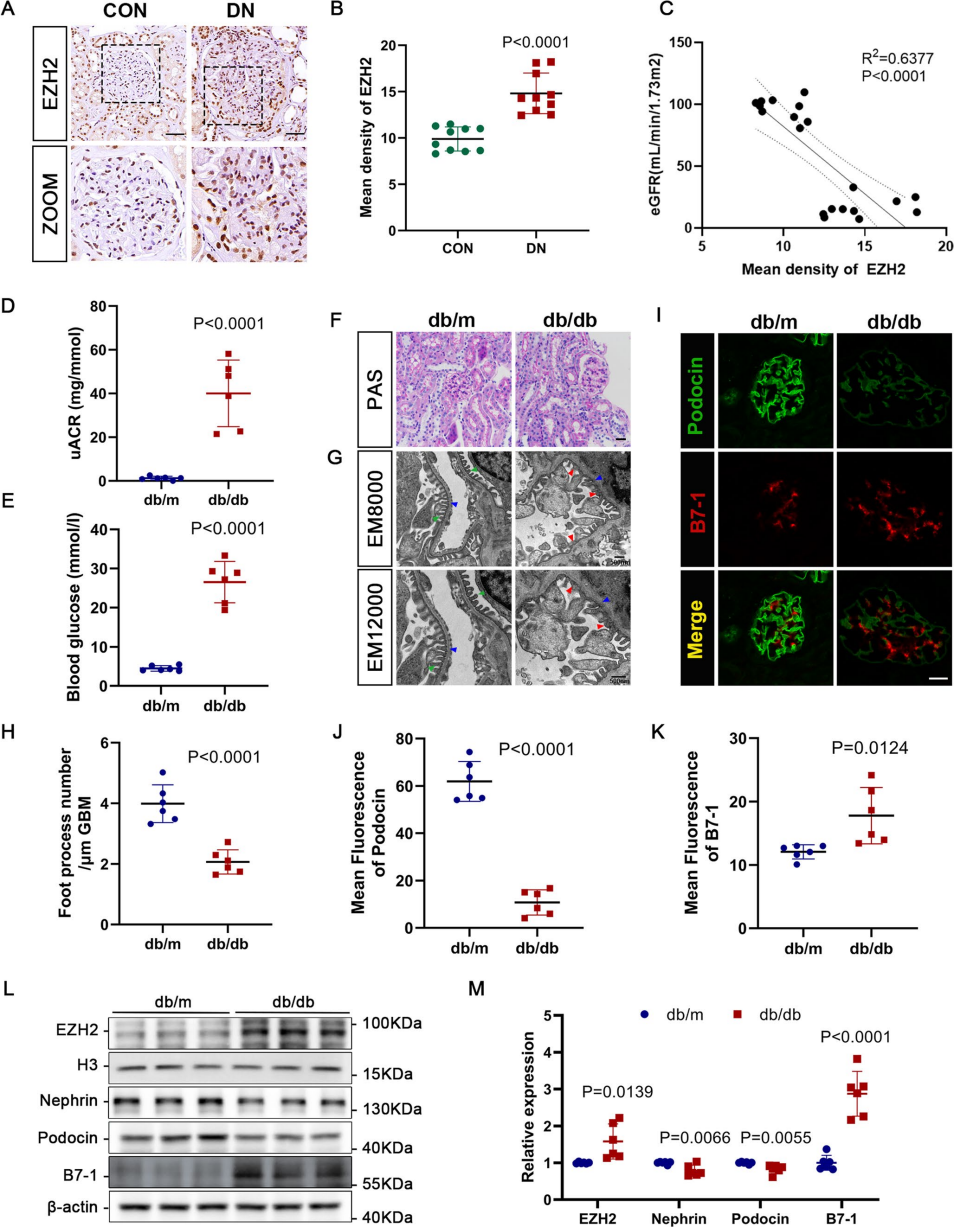
Expression of EZH2 and podocyte injury were enhanced in db/db mice. (**A**-**B**) Representative images and quantitative analysis of immunohistochemistry staining of EZH2 in the glomeruli of human renal samples (40×). Scale bar = 50 μm. *P < 0.05 vs. control group (*n* = 10). (**C**) Correlation analysis between estimated glomerular filtration rate (eGFR) and the expression of EZH2 in DN patients. (**D**-**E**) Urinary albumin/creatine ratios (uACRs) and blood glucose levels measured in each group. *P* values vs. db/m group (*n* = 6). (**F**) Representative images of periodic acid-Schiff (PAS) staining revealing glomerular alterations in each group (40×). Scale bar = 20 μm. (**G**) Micrographs of podocyte foot processes (green arrows), podocyte foot process effacement and fusion (red arrows) and glomerular basement membrane (GBM, blue arrows) observed by transmission electron microscopy (TEM) in each group. Scale bar = 500 nm. (**H**) Quantification of the number of foot processes in different groups of mice. *P* values vs. db/m group (*n* = 6). (**I**-**K**) Illustrative confocal microscopic images and quantifications of podocin (green) and B7-1 (red) in each group. Scale bar = 20 μm. *P* values vs. db/m group (*n* = 6).** (L-M)** Western blotting and quantitative analysis of EZH2, nephrin, podocin, and B7-1 levels in isolated glomeruli in each group. Histone 3 (H3) and β-actin served as loading controls. *P* values vs. db/m group (*n* = 6). CON, control; DN, diabetic nephropathy

According to the single-cell RNA sequencing analysis of EZH2 expression in different cell types obtained from the Kidney Single Cell online analysis database (Kidney Interactive Transcriptomics, http://humphreyslab.com), EZH2 was significantly up-regulated in DN patients, especially in podocytes (Fig. S1) [22, 23]. The immunohistochemical staining showed an increase in the expression of EZH2 in glomeruli of DN patients compared to the control group (Fig. 1A, B). Furthermore, the levels of EZH2 in the glomeruli existed a negative correlation with estimated glomerular filtration rate (eGFR, Fig. 1C). Therefore, in this study, we mainly focused on the role of EZH2 in podocytes in DN.

Initially, we established leptin receptor-deficient db/db mice exhibiting spontaneous type 2 DM as an in vivo model of DN, which manifesting a rapidly increase of blood glucose starting 4-week-age and appearing proteinuria from 12-week-age. Elevated urinary albumin/creatinine (uACR) and blood glucose levels were observed in 22-week db/db mice compared to db/m mice, suggesting that the presence of renal injury in db/db mice (Fig. 1D, E). PAS staining also revealed the increased mesangial matrix deposition and glomerular hypertrophy in the glomeruli of db/db mice (Fig. 1F). Furthermore, podocyte foot process effacement and thickened GBM were observed in db/db mice using TEM (Fig. 1G, H). These alterations indicated the impairment of kidneys in the db/db mice.

Then, we detected the levels of EZH2, podocyte markers (nephrin and podocin), and a podocyte inflammation factor (B7-1, a critical mediator that induces podocyte injury and glomerulosclerosis) in the glomeruli extracted from each mouse [24]. Our study showed that EZH2 was upregulated in db/db mice compared to db/m mice, as indicated by western blotting (Fig. 1L, M). Moreover, podocyte marker protein levels were reduced, and B7-1 was increased in db/db mice, which was consistent with the injury shown in the micrographs (Fig. 1L, M). Likewise, immunofluorescence staining further emphasized that podocyte injury was more prominent in the glomeruli of db/db mice (Fig. 1I-K). Consequently, our results indicated the elevated EZH2 levels and enhanced podocyte injury in DN.

### Increased EZH2 and podocyte injury in HG-induced podocytes

**Fig. 2:**
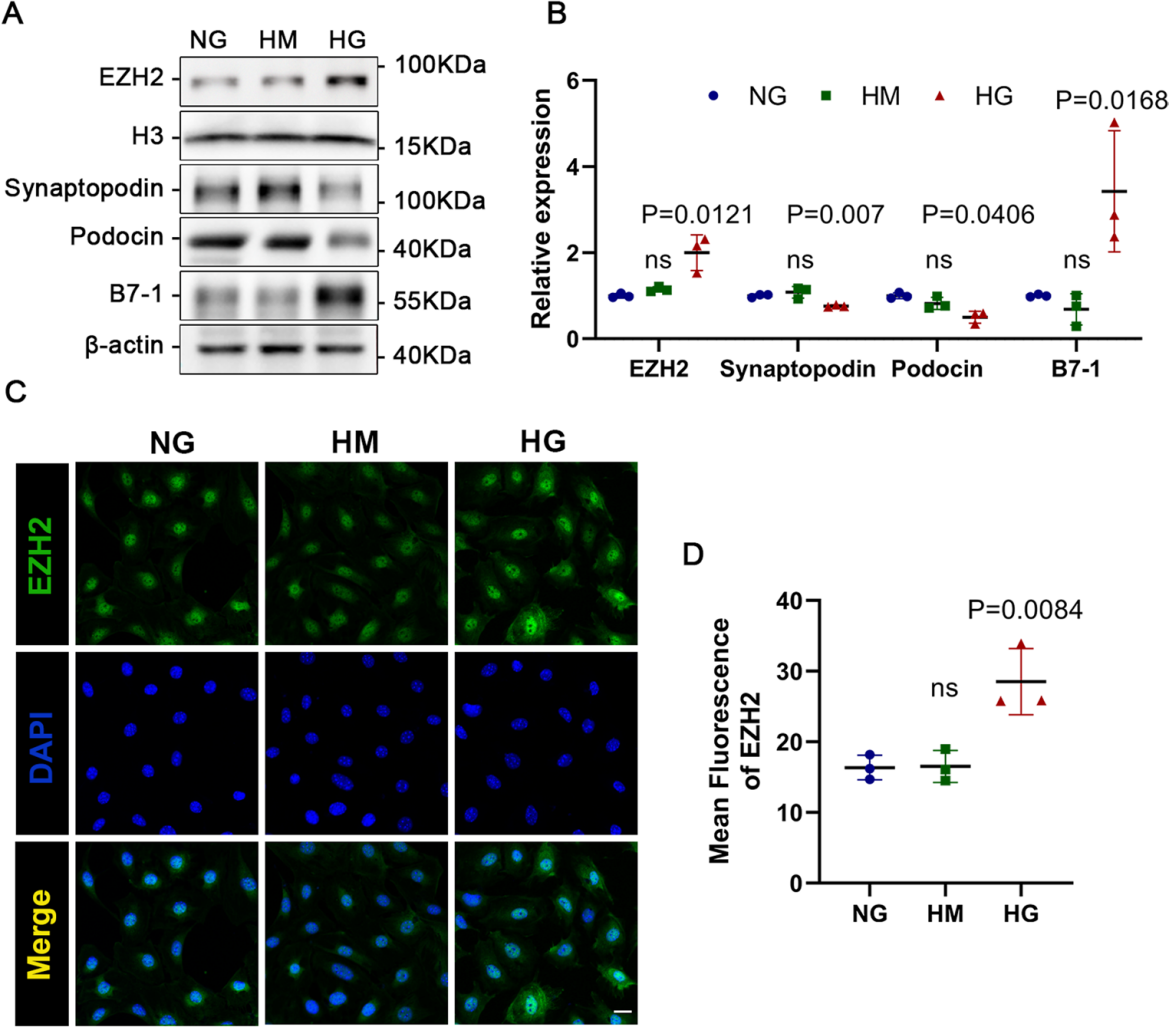
Up-regulated EZH2 levels and aggravated podocyte injury in high glucose-cultured MPCs. (**A**-**B**) Western blotting and quantitative analysis of EZH2, synaptopodin, podocin, and B7-1 levels in mouse podocyte cells (MPCs). H3 and β-actin served as loading controls. ns: *P* > 0.05 vs. NG group. *P* values vs. NG group (*n* = 3). (**C**-**D**) Illustrative confocal microscopic images and quantification showed EZH2 (green) in the MPCs counterstained with 4’,6-diamidino-2-phenylindole (DAPI) (blue). Scale bar = 20 μm. ns: *P* > 0.05 vs. NG group. *P* values vs. NG group (*n* = 3). NG, normal glucose; HM, high mannitol; HG, high glucose

Next, in order to investigate alterations involving EZH2 expression and podocyte injury status under diabetic conditions, we utilized HG-stimulated MPCs as an in vitro DN model. Similarly, the expression of EZH2 in HG-stimulated MPCs was significantly higher compared to the control groups (NG group and HM group) (Fig. 2A, B). This was corroborated by immunofluorescence staining, which indicated enhanced EZH2 expression in HG-induced podocytes (Fig. 2C, D). In addition, compared to the control groups, the levels of podocyte-specific markers (synaptopodin and podocin) were decreased, whereas B7-1 was elevated in the HG group (Fig. 2A, B). In summary, the above results indicated an elevation in the levels of EZH2 expression and podocyte injury in DN.

### Cellular senescence is accelerated in DN

Multiple studies have provided evidence suggesting that podocyte senescence may play a significant role in DN [25]. Klotho, an anti-aging protein and specific biomarker of renal tissue senescence, has been demonstrated to alleviate histological lesions and preserve renal function in DN by inducing anti-inflammatory responses, inhibiting oxidative stress, and reducing leukocyte infiltration and renal fibrosis [26, 27]. Moreover, inhibiting the sustained expression of p21, a biomarker of aging, can reverse renal hyperglycemic memory in DN [28]. Considering these findings, we examined the expression of Klotho and p21 as indicators of cellular senescence in our DN models.

**Fig. 3:**
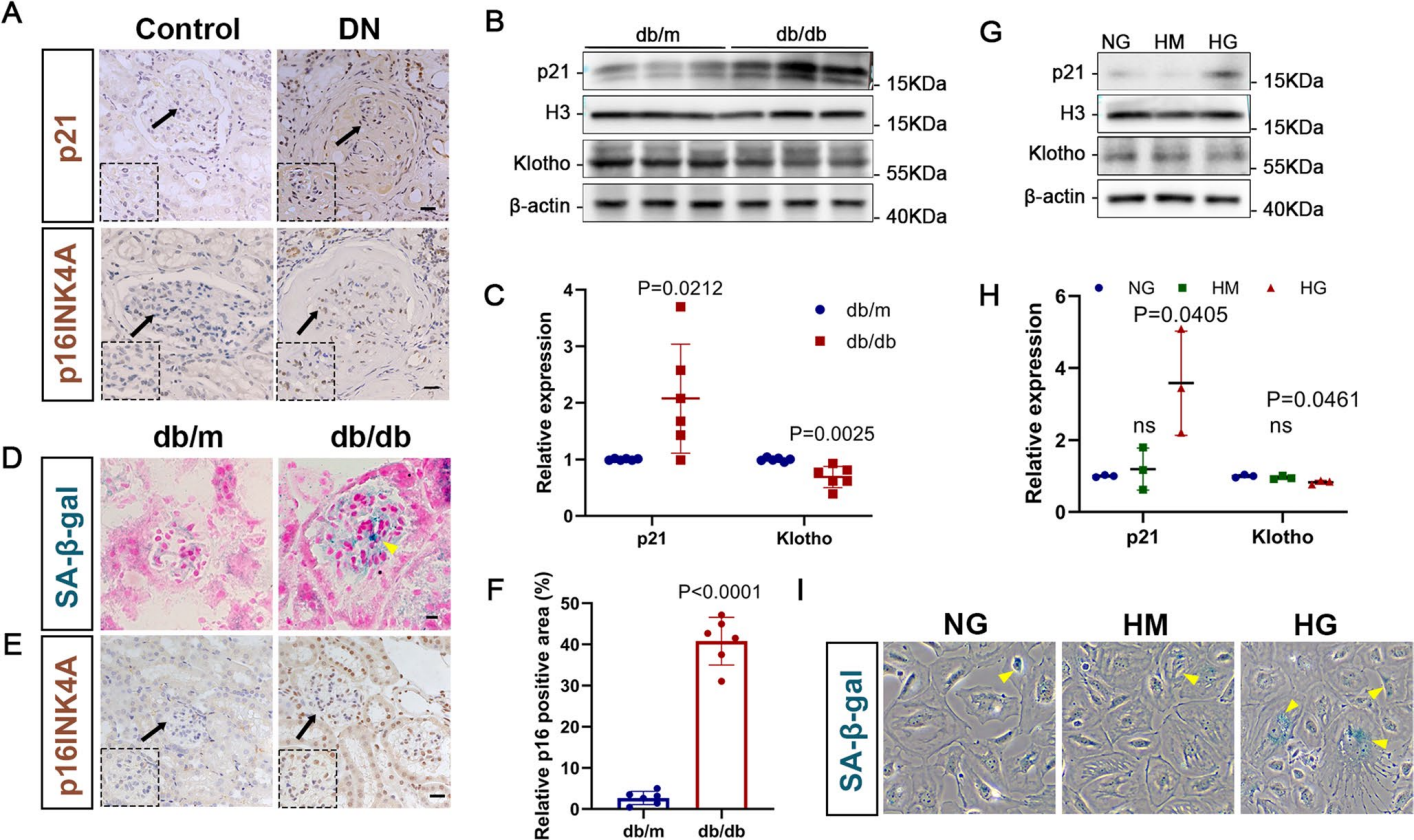
Cellular senescence is accelerated in DN. (**A**) Representative graphs of p21 and p16^INK4A^ in DN patients’ renal tissues as detected by immunohistochemistry staining (40×). Scale bar = 20 μm. The dashed lines represent the enlarged images of the area indicated by the arrows. (**B**-**C**) Western blotting and quantitative analysis of p21 and Klotho levels in each group. H3 and β-actin served as loading controls. *P* values vs. db/m group (*n* = 6). (**D**-**E**) Graphs of senescence-associated β-galactosidase (SA-β-gal, yellow arrows) staining and immunohistochemistry staining for p16^INK4A^ in each group (40×). Scale bar = 20 μm. The dashed lines represent the enlarged images of the area indicated by the arrows. (**F**) Quantitative analysis of p16^INK4A^ in the groups of mice. *P* values vs. db/m group (*n* = 6). (**G**-**H**) Western blotting and quantitative analysis of p21 and Klotho in MPCs. H3 and β-actin served as loading controls. ns: *P* > 0.05 vs. NG group. *P* values vs. NG group (*n* = 3). (I) Graphs of SA-β-gal (yellow arrows) staining among MPCs

We first verified that p21 and p16^INK4A^ expression were augmented in renal biopsy specimens from patients with DN (Fig. 3A). Next, an increase in p21 and a decline in Klotho protein levels were observed in db/db mice compared to db/m mice (Fig. 3B, C). In addition, glomeruli from db/db mice exhibited a greater area of SA-β-gal-positive staining (Fig. 3D). Moreover, the expression of p16^INK4A^ in the glomeruli of db/db mice was increased (Fig. 3E, F). Similarly, podocytes treated with HG showed lower expression of Klotho and higher expression of p21 compared to the control groups (Fig. 3G, H). Furthermore, the number of senescent podocytes was increased in the HG group compared to the control groups, as evidenced by SA-β-gal staining (Fig. 3I). These findings collectively indicate a more pronounced podocyte senescence in DN.

### Pharmacological Inhibition of EZH2 alleviates podocyte injury and delays glomerular senescence in db/db mice

**Fig. 4:**
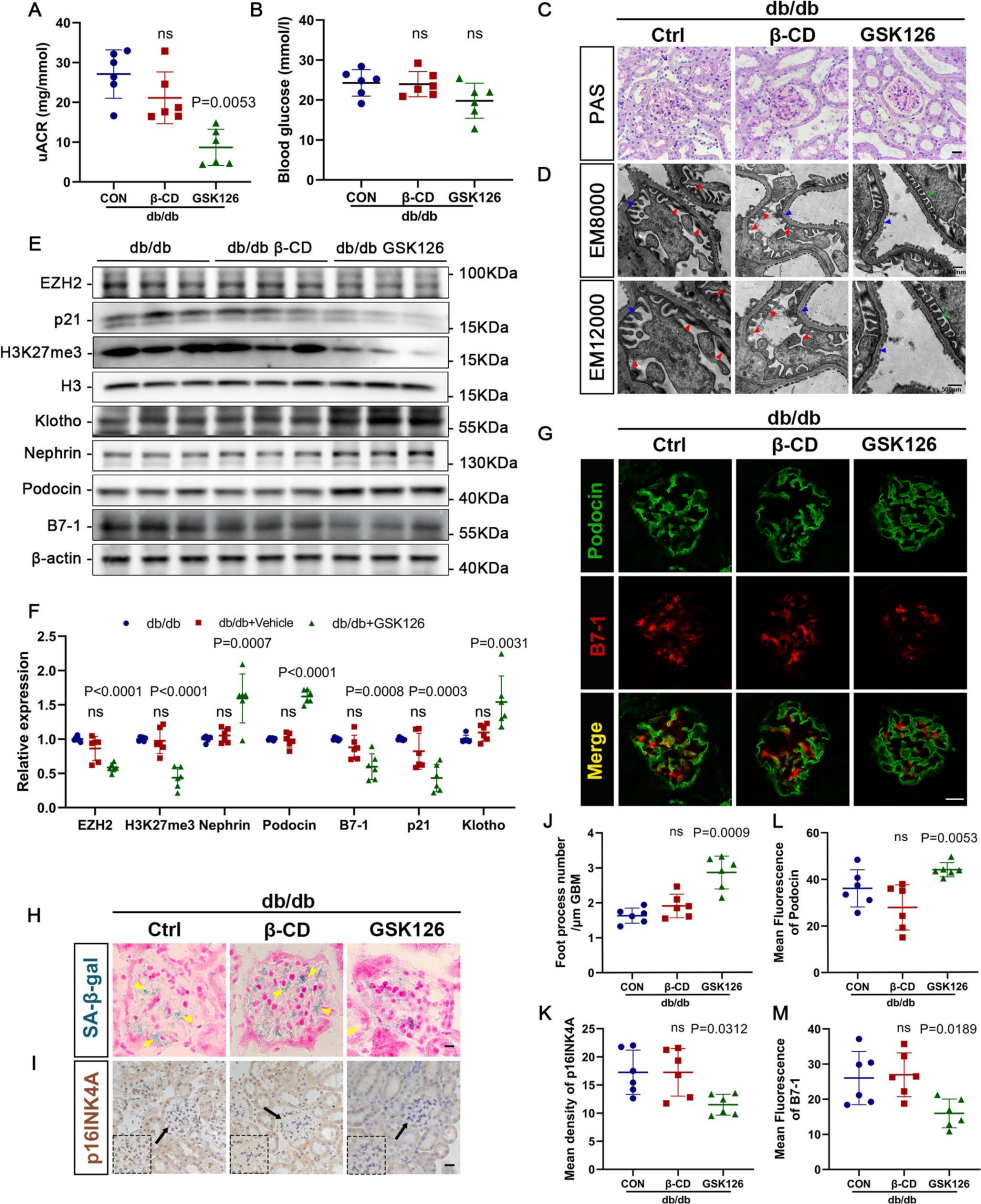
The EZH2 inhibitor GSK126 alleviated podocyte injury and glomerular senescence in db/db mice. (**A**-**B**) uACRs and blood glucose levels measured in each group. ns: *P* > 0.05 vs. db/db group. *P* values vs. db/db group (*n* = 6). (**C**) Representative images of PAS staining of glomeruli in each group (40×). Scale bar = 20 μm. (**D**) Micrographs of podocyte foot processes (green arrows), podocyte foot process effacement and fusion (red arrows) and GBM (blue arrows) observed by TEM in each group. Scale bar = 500 nm. (**E**-**F**) Western blotting and quantitative analysis of EZH2, H3K27me3, nephrin, podocin, B7-1, p21, and Klotho in each group. H3 and β-actin served as loading controls. ns: *P* > 0.05 vs. db/db group. *P* values vs. db/db group (*n* = 6). (**G**) Illustrative confocal microscopic images of podocin (green) and B7-1 (red) in different groups. Scale bar = 20 μm. (**H**-**I**) Graphs of SA-β-gal (yellow arrows) staining and immunohistochemistry staining for p16^INK4A^ in each group (40×). Scale bar = 20 μm. The dashed lines represent the enlarged images of the area indicated by the arrows. (**J**-**K**) Quantifications of the number of foot processes and p16^INK4A^ in different groups of mice. ns: *P* > 0.05 vs. db/db group. *P* values vs. db/db group (*n* = 6). (**L**-**M**) Quantitative analysis of podocin and B7-1 in the glomeruli of different groups. ns: *P* > 0.05 vs. db/db group. *P* values vs. db/db group (*n* = 6). β-CD, SBE-β-CD; GSK126, GSK2816126A

To determine the relationship between EZH2 and aggravated podocyte injury and senescence in DN, we used GSK126, a S-adenosylmethionine competitive inhibitor that selectively targets EZH2, to restrain the expression of EZH2 in db/db mice. We injected mice with SBE-β-CD (50 mg/kg) or GSK126 (50 mg/kg) intraperitoneally for 5-days per week over 8-weeks. Biochemical indicators revealed that, compared to control mice (db/db and db/db + β-CD mice), mice injected with GSK126 exhibited a trend towards decreased uACR, but no significant difference in blood glucose levels (Fig. 4A, B). In addition, PAS staining showed that db/db mice exposed to GSK126 presented less severe mesangial matrix deposition and glomerular hypertrophy than control mice (Fig. 4C). Furthermore, podocyte foot process effacement and GBM thickening were alleviated by GSK126 administration, as determined by TEM (Fig. 4D, J).

Western blotting corroborated the above findings, indicating that podocyte-specific markers (nephrin and podocin) were recovered in db/db + GSK126 mice compared to those in control mice (Fig. 4E, F). Meanwhile, levels of inflammatory factor B7-1 also declined concomitant with EZH2 inhibition (Fig. 4E, F). Immunofluorescence staining revealed consistent results (Fig. 4G, L, M). Moreover, the upregulation of p21 and downregulation of Klotho in db/db mice were reversed by GSK126, as evidenced in Fig. 4E, F. Additionally, SA-β-gal staining also demonstrated a decreased accumulation of β-galactosidase in the glomeruli of mice treated with GSK126 (Fig. 4H). Similarly, EZH2 inhibition also reduced the expression of p16^INK4A^ in db/db mice (Fig. 4I, K). These findings indicated that EZH2 inhibition attenuates the development of podocyte damage and glomerular senescence in DN.

### STAT3 is methylated and activated through its interaction with EZH2

**Fig. 5:**
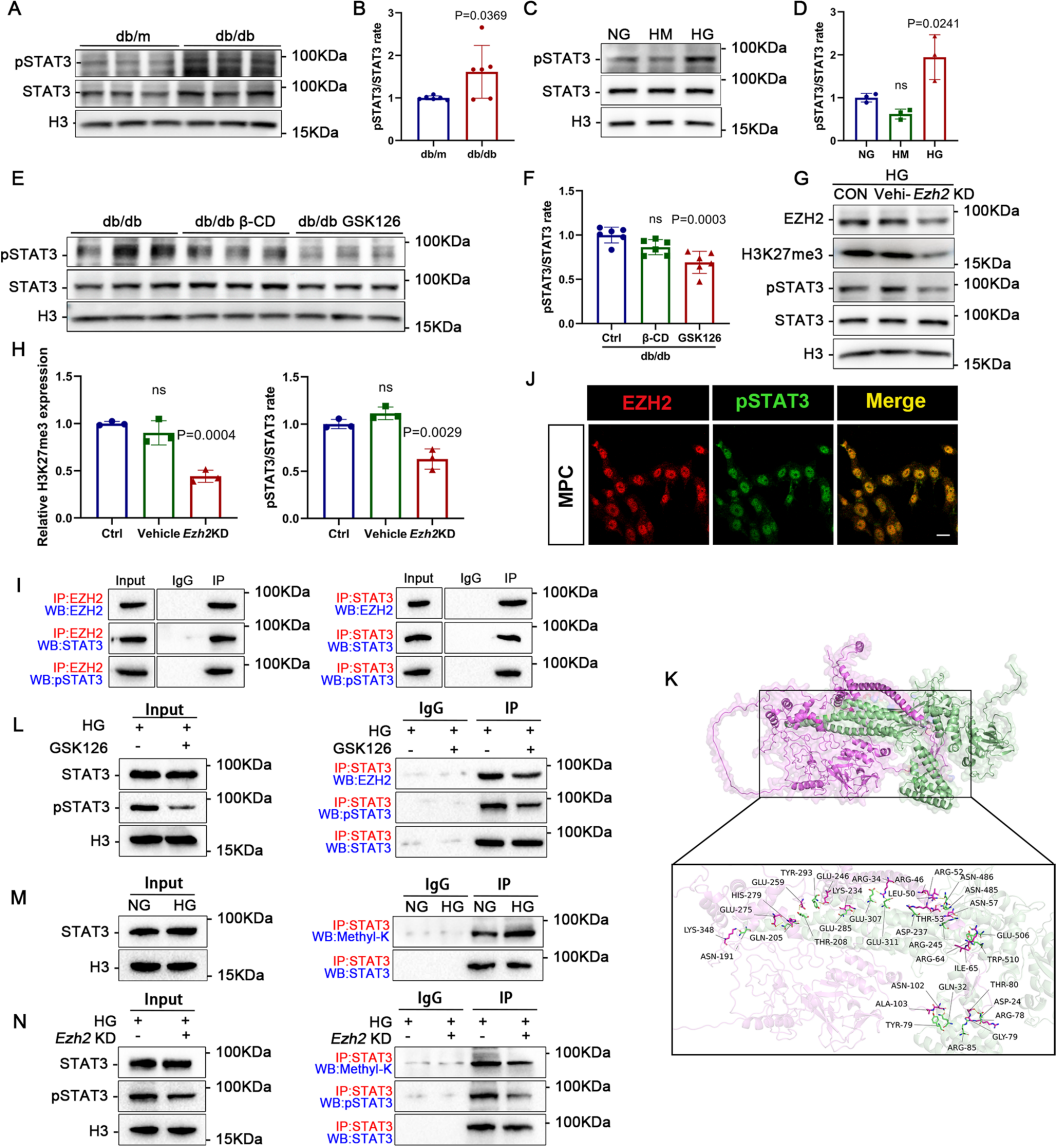
EZH2 methylates and activates STAT3 in podocytes. (**A**-**B**) Western blotting and quantitative analysis of pSTAT3/STAT3 in each group. *P* values vs. db/m group (*n* = 6). (**C**-**D**) Western blotting and quantitative analysis of pSTAT3/STAT3 in MPCs. ns: *P* > 0.05 vs. NG group. *P* values vs. NG group (*n* = 3). (**E**-**F**) Western blotting and quantifications of pSTAT3/STAT3 in different groups. ns: *P* > 0.05 vs. db/db group. *P* values vs. db/db group (*n* = 6). (**G**-**H**) Western blotting and quantitative analysis of pSTAT3/STAT3 and H3K27me3 in MPCs. ns: *P* > 0.05 vs. HG group. *P* values vs. HG group (*n* = 3). (**I**) Co-immunoprecipitation (Co-IP) revealed a relationship between EZH2 and STAT3, as well as EZH2 and pSTAT3. The Input group served as a positive control, and the IgG group served as a negative control. (**J**) Illustrative confocal microscopic images of EZH2 (red) and pSTAT3 (green) in MPCs counterstained with DAPI (blue). Scale bar = 20 μm. (**K**) Image of molecular docking between EZH2 (pink) and STAT3 (green). (**L**) Co-IP showed the interaction between EZH2 and STAT3 after EZH2 inhibition by GSK126. (**M**) The methylation status of lysine residues of STAT3 was evaluated by Co-IP in MPCs treated with NG or HG. (**N**) The methylation status of lysine residues of STAT3 after EZH2 knockdown (KD) by adenovirus

Recent studies have reported that EZH2 can combine with STAT3 and subsequently enhance its activity by increasing STAT3 methylation via interprotein interactions [16]. Here, as STAT3 is also a key mediator in the pathogenesis of various renal diseases, we assessed the levels of total STAT3 and its phosphorylation status in murine DN models [29]. Compared to db/m mice, the pSTAT3/STAT3 ratio was markedly upregulated in db/db mice (Fig. 5A, B). Consistently, an increased pSTAT3/STAT3 ratio was observed in HG-treated MPCs compared to the control groups (Fig. 5C, D). Furthermore, db/db mice injected with GSK126 exhibited lower levels of pSTAT3/STAT3 compared to control mice (Fig. 5E, F). Next, we used an adenovirus to knockdown EZH2 expression in HG-treated MPCs (Fig. S2). Likewise, the pSTAT3/STAT3 ratio was decreased after EZH2 blocking (Fig. 5G, H). These results indicated that EZH2 was correlated to STAT3 activity.

To identify whether EZH2 interact with STAT3 in MPCs under protein level, Co-IP experiments were performed. As expected, the results of bidirectional Co-IP showed that the binding between EZH2 and STAT3 in cultured MPCs were positive (Fig. 5I). Interestingly, there was also an interaction and co-localization between EZH2 and pSTAT3 (Fig. 5I-J). In addition, the molecular docking also indicated a binding between EZH2 and STAT3 (Fig. 5K). We also found that inhibiting EZH2 by GSK126 reduced the interaction between EZH2 and STAT3 (Fig. 5L). Notably, the phosphorylation of STAT3 was also reduced after EZH2 inhibition (Fig. 5L, N). Subsequently, we measured the levels of pan-methylated lysine in STAT3 in cultured MPCs to examine if the methylation of STAT3 has changed by EZH2. The methylation status of STAT3 increased after HG stimulation compared to the NG group (Fig. 5M), and this enhanced methylation of STAT3 was reversed via EZH2 suppression by EZH2 KD adenovirus (Fig. 5N). These results further demonstrate that EZH2 could bind to STAT3 under protein-protein interactions and activate STAT3 by enhancing the methylation levels of its lysine residue.

### Renoprotective effects of EZH2 Inhibition can be reversed by STAT3 overexpression in db/db mice

**Fig. 6:**
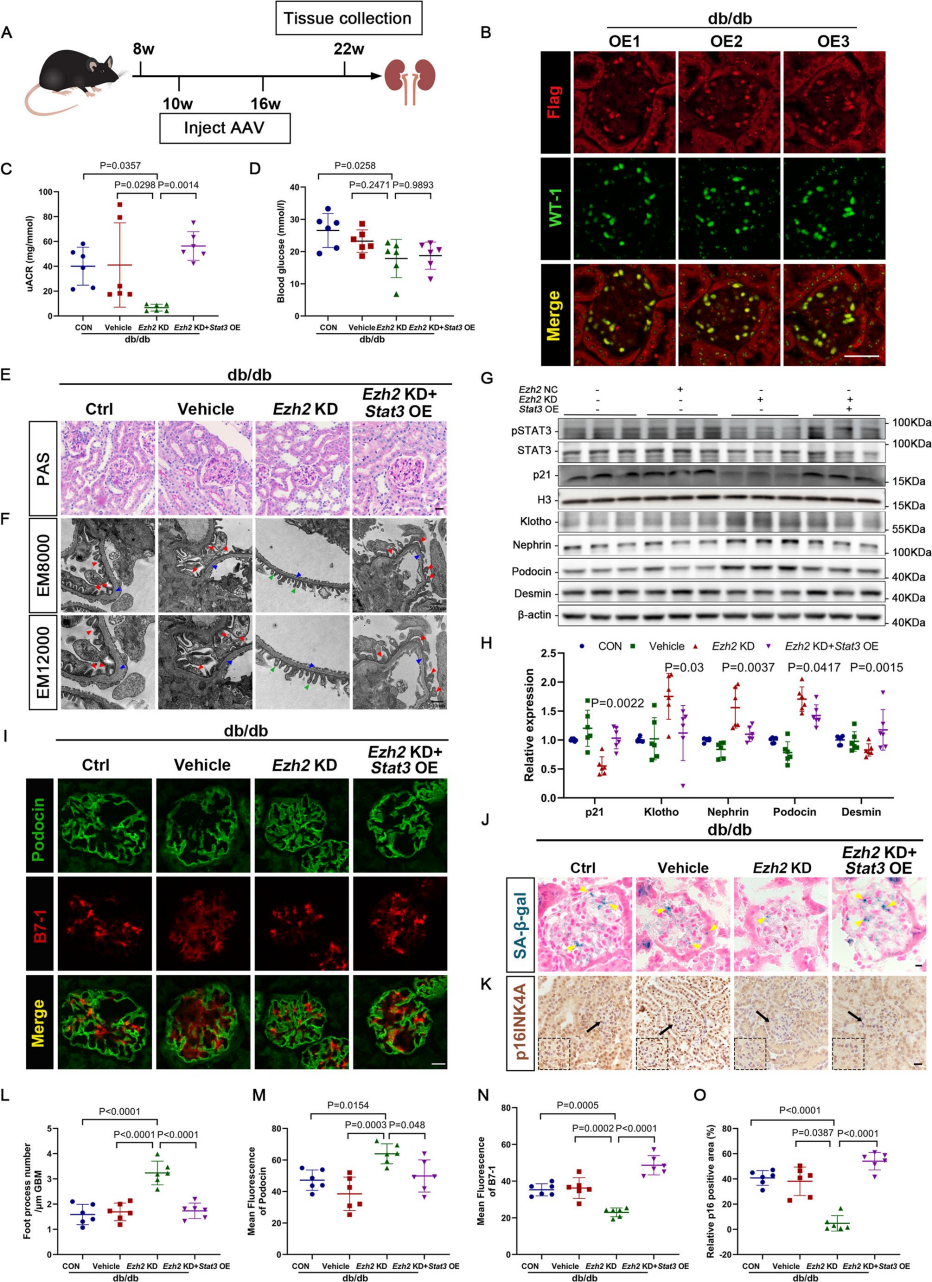
Overexpression of STAT3 eliminated the reversal role of EZH2 inhibition against podocyte damage and senescence in db/db mice. (**A**) Flow chart of treatment, sampling and observation end points for the db/db mice. (**B**) Illustrative immunofluorescence staining images of Flag (red) and Wilms Tumor-1 (green) in db/db + EZH2 KD + STAT3 OE group mice. Scale bar = 20 μm. (**C**-**D**) uACRs and blood glucose levels were measured in different groups (*n* = 6). (**E**) Representative images of PAS staining of glomeruli in each group (40×). Scale bar = 20 μm. (**F**) Micrographs of podocyte foot processes (green arrows), podocyte foot process effacement and fusion (red arrows) and GBM (blue arrows) as observed by TEM in each group. Scale bar = 500 nm. (**G**-**H**) Western blotting and quantitative analysis of p21, Klotho, nephrin, podocin, and desmin levels in each group. H3 and β-actin served as loading controls. *P* values vs. db/db + EZH2 KD group (*n* = 6). (**I**) Illustrative confocal microscopic images of podocin (green) and B7-1 (red) in each group. Scale bar = 20 μm. (**J**-**K**) Graphs of SA-β-gal (yellow arrows) staining and immunohistochemistry staining for p16^INK4A^ in each group (40×). Scale bar = 20 μm. The dashed lines represent the enlarged images of the area indicated by the arrows. (**L**-**O**) Quantifications of the number of foot processes, podocin, B7-1 and p16^INK4A^ in different groups of mice (*n* = 6). OE, overexpression

To validate whether the adverse effect of EZH2 on podocytes was mechanistically mediated by activation of STAT3 phosphorylation, *NPHS2*-promoter recombinant AAV to achieve podocyte-specific EZH2 knockdown and STAT3 overexpression were delivered into the kidneys of db/db mice via tail-vein injection (Fig. 6A). The transfection efficiency of STAT3-overexpressing AAV is shown in Fig. 6B. At the end of the observation period, the uACR in db/db mice was decreased by EZH2 inhibition, whereas the advantageous renoprotective role of EZH2 KD was abolished by overexpression of STAT3 in db/db mice (Fig. 6C). However, no remarkable differences were found in blood glucose levels among the groups (Fig. 6D). PAS staining showed that glomerular injury was reduced in db/db + EZH2 KD mice but aggravated in mice following EZH2 KD + STAT3 OE (Fig. 6E). TEM also indicated that EZH2 KD ameliorated podocyte foot process effacement and GBM thickening in db/db mice, whereas podocyte lesions were exacerbated in the db/db + EZH2 KD + STAT3 OE group (Fig. 6F, L).

Additionally, the levels of podocyte markers (nephrin and podocin) were restored, and desmin expression was decreased in db/db + EZH2 KD mice, while STAT3 OE elicited the opposite effect, as evidenced by western blotting (Fig. 6G, H). Immunofluorescent staining also showed that podocyte injury was aggravated in db/db + EZH2 KD + STAT3 OE mice compared to db/db + EZH2 KD animals (Fig. 6I, M, N). Moreover, the expression of p21 was reduced, while that of Klotho was increased in db/db mice with EZH2 downregulation, and STAT3 overexpression reversed these changes (Fig. 6G, H). Consistently, the positive area of SA-β-gal staining was decreased in db/db + EZH2 KD mice, while the same was increased with the overexpression of STAT3 (Fig. 6J). In addition, expression of p16^INK4A^ was increased in db/db + EZH2 KD + STAT3 OE mice compared to db/db + EZH2 KD mice, as determined by immunohistochemistry staining (Fig. 6K, O). Overall, the EZH2/STAT3 axis appears to mechanistically regulate podocyte injury and glomerular senescence in db/db mice, at least to a certain degree.

### Overexpression of STAT3 counteracts beneficial effects of EZH2 Inhibition in podocytes

**Fig. 7:**
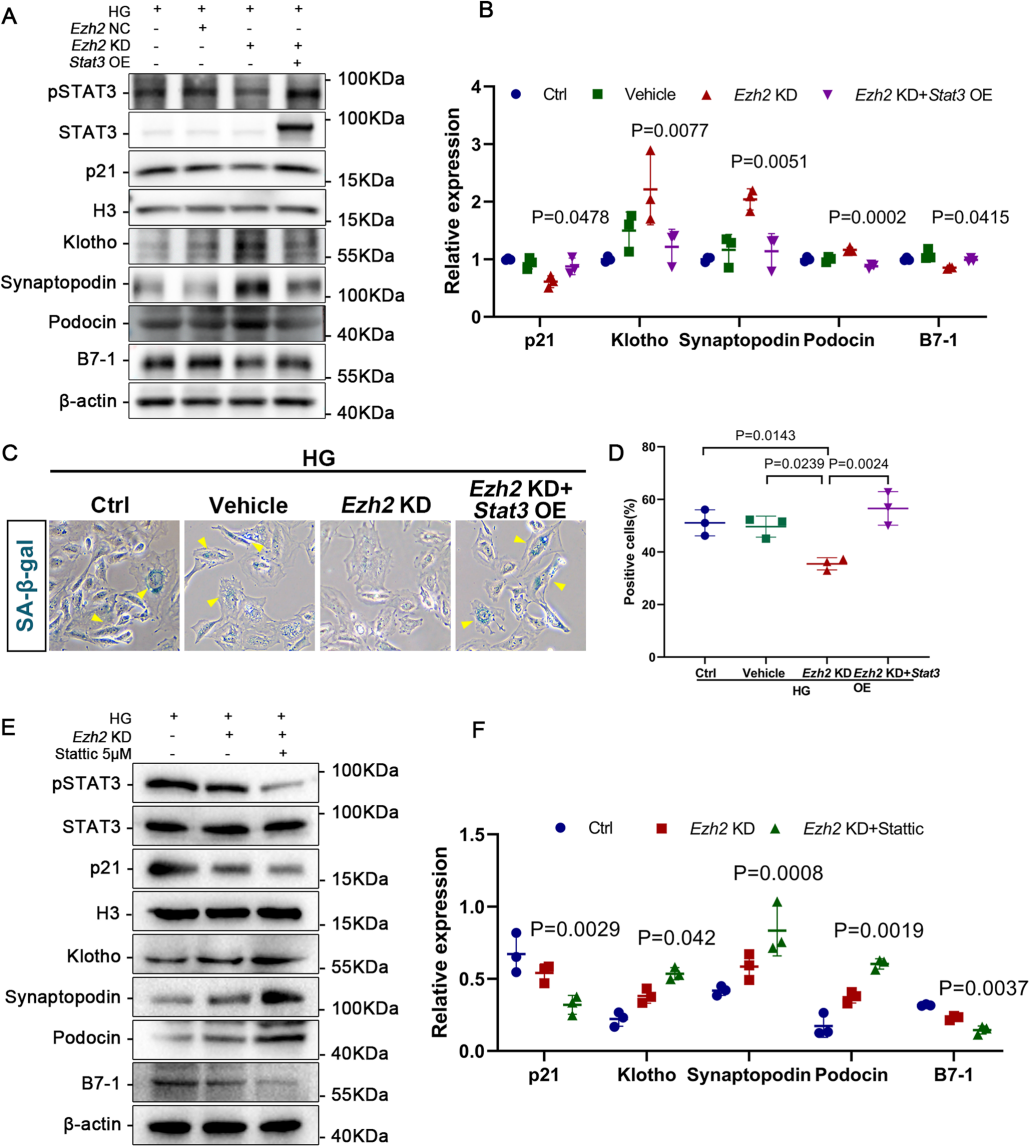
STAT3 overexpression counteracts podocyte protection mediated by EZH2 inhibition in high glucose-treated MPCs. (**A**-**B**) Western blotting and quantitative analysis of p21, Klotho, synapotopodin, podocin, and B7-1 levels. H3 and β-actin served as loading controls. *P* values vs. HG + EZH2 KD group (*n* = 3). (**C**-**D**) Graphs and quantitative analysis of SA-β-gal (yellow arrows) staining in MPCs (*n* = 3). (**E**-**F**) Representative images of western blotting and quantifications of p21, Klotho, synapotopodin, podocin, and B7-1 levels. H3 and β-actin served as loading controls. *P* values vs. HG + EZH2 KD group (*n* = 3)

**Fig. 8:**
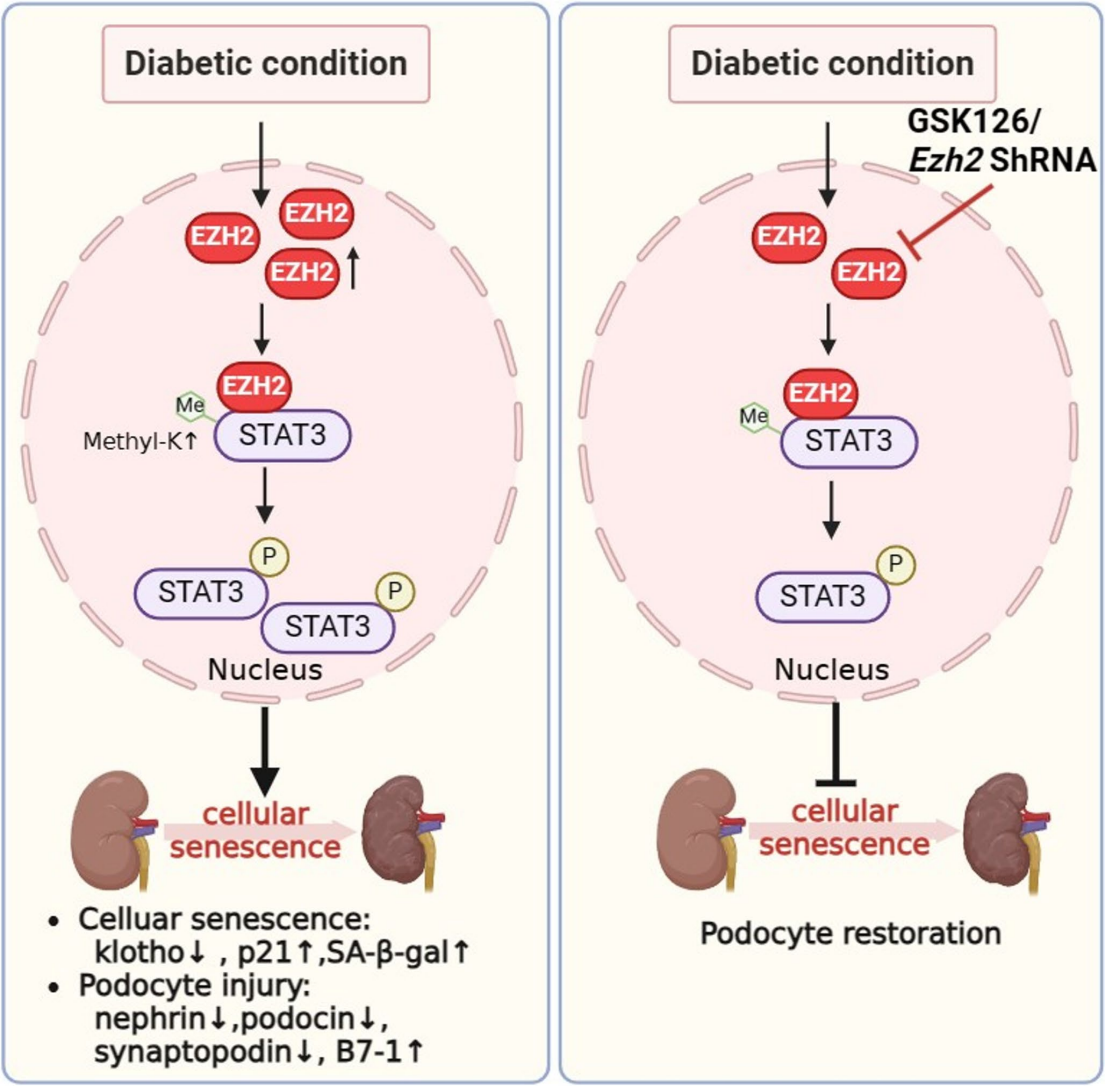
A schematic diagram illustrates the multifaceted role of the EZH2 in podocyte senescence upon diabetic insult and the protective effect of targeting EZH2. The present study demonstrated that EZH2 is highly expressed in DN and negatively correlated with eGFR. EZH2-mediated STAT3 activation promotes podocyte senescence. The aforementioned pathogenic pathways contribute to kidney aging in DN and can be mitigated by targeting EZH2, either genetically through adeno-associated virus-mediated knockdown, or pharmacologically using small-molecule inhibitors such as GSK126

Next, we performed rescue experiment on the cultured podocytes. The expression of synaptopodin and podocin was diminished, while that of inflammatory factor B7-1 was elevated in the HG + EZH2 KD + STAT3 OE group compared to the EZH2 KD group (Fig. 7A, B). Compared with the HG + EZH2 KD group, p21 was increased and Klotho was reduced in the HG + EZH2 KD + STAT3 OE group (Fig. 7A, B). In addition, the percent of SA-β-gal positive podocytes was decreased in podocytes under EZH2 inhibition, while overexpression of STAT3 increased numbers of SA-β-gal positive cells (Fig. 7C, D). Interestingly, the beneficial effects in MPCs produced by EZH2 KD were enhanced by treatment with stattic, an inhibitor of STAT3 (Fig. 7E, F). Above results demonstrated that the function of EZH2 in podocyte injury and senescence is mediated via STAT3 activation (Fig. 8).

## Discussion

Podocytes, together with endothelial cells and the GBM, construct glomerular filtration barrier and restrict the passage of proteins depending on high electronegativity and the slit diaphragm formed by the long and regularly interdigitated foot processes [30, 31]. Podocyte injury represents an early manifestation of glomerular injury in DN, which can arise from various cell stresses and induce proteinuria and glomerular sclerosis [32]. Therefore, it is of great significance to elucidate the mechanisms involved in podocyte injury in DN.

EZH2 is a crucial epigenetic regulator and catalyzes the H3K27me3 mark, and thereby leading to gene silencing by the recruitment of EZH2/H3K27me3 mark to target gene promoters [33]. A large body of evidence has shown that abnormal expression of EZH2/H3K27me3 is related to various kidney diseases, including DN [13, 23, 34]. Previous studies have shown that EZH2 expression in kidney tissues and plasma of DN patients significantly exceeds that of control group [34, 35]. Moreover, levels of EZH2 in kidney tissues are positively correlated with serum creatine and, 24-h proteinuria, and negatively correlated with eGFR in DN patients [34]. In the early stages of the disease in db/db mice, EZH2 upregulation promotes mesangial cell hypertrophy, stromal cell expansion, and fibrotic protein increase by inhibiting Deptor and activating the mammalian target of rapamycin signal [36]. In their study, Tang et al. confirmed that celastrol could restrain EZH2, thereby alleviating podocyte injury, apoptosis, and oxidative stress and reducing proteinuria and renal histological changes in db/db mice [37]. Furthermore, a study by Wan et al. also demonstrated that in STZ-induced diabetic rats, silencing EZH2 can inhibit the Wnt/β-catenin signaling, thereby reducing podocyte oxidative stress and apoptosis and increasing podocyte integrity [34]. Our previous study has identified that lncRNA *PVT1* can inhibit FOXA1 expression by recruiting EZH2, thereby promoting podocyte injury and apoptosis in DN [38]. Given EZH2’s complex roles, we established DN models to investigate its functions and potential mechanisms in podocytes. Our data suggested that EZH2 was negatively correlated with eGFR in human kidneys. Pharmacological inhibition of EZH2 by GSK126 could reverse the decrease in podocyte-specific biomarkers and increase inflammatory factors in db/db mice. EZH2 suppression also ameliorated proteinuria and glomerular damage in db/db mice, findings which are consistent with previous studies’ results.

Nevertheless, the role of EZH2 in podocyte injury in DN remains controversial. It has been reported that podocytes lacking H3K27me3 renders mice more susceptible to glomerular disease [39]. However, whether this effect is due to EZH2 depletion or upregulated histone demethylase of H3K27me3 is still debatable. Siddiqi et al. demonstrated that inhibiting EZH2 with 3-deazaneplanocin increases podocyte injury, oxidative stress, and proteinuria in diabetic rats [40]. 3-deazaneplanocin is a nonspecific histone methyltransferase (HMT) inhibitor that can also inhibit the activity of other HMTs in addition to EZH2 [41]. In contrast, the inhibitor GSK126 used in our study is a highly selective inhibitor of EZH2 [42]. Therefore, further studies are required to clarify EZH2’s role in podocytes in DN.

Cellular senescence involves the irreversible arrest of the cell cycle, which is a complicated process involving various mechanisms, including DNA damage, telomere shortening, inflammation, oxidative stress, and epigenetics [7]. The diabetic environment can accelerate senescence, and in turn, senescence accelerates the development of diabetic complications [43]. Interestingly, the alleviation of podocyte senescence also plays a crucial role in preventing DN progression [25]. Elevated p16 expression and SA-β-gal activity have been observed in podocytes in both type 2 DM patients and STZ-induced type 1 diabetic mice [9]. The age-associated decline in Klotho expression by renal tubular cells was partly attributed to upregulated levels of H3K27me3, suggesting that EZH2 may play a vital role in renal senescence [44]. Moreover, a recent study by Wen et al. found that EZH2 can accelerate tubular senescence in cisplatin-induced acute kidney injury through mutual regulation of the aryl hydrocarbon receptor [13]. In our study, levels of the anti-aging protein Klotho decreased, while the p21 expression increased in DN models. Our results also indicated that the proportions of senescent podocytes under HG environment and the positive areas of SA-β-gal staining in glomeruli of db/db mice were influenced by EZH2 levels. Repressing EZH2 with GSK126 exposure can ameliorate glomerular senescence in db/db mice.

Previous studies have shown that aside from its canonical role in methylating H3, EZH2 can directly methylate other various proteins (e.g. androgen receptor and GATA4) and regulate the transcriptional activity of substrates in a PRC2-dependent or independent manner without participation of H3K27me3, thereby mediating the occurrence and development of diseases [16, 17]. It is notable that EZH2 can bind to STAT3 directly and stimulate STAT3 methylation and activity via a non-histone methylation pathway in glioblastoma stem-like cells [16]. STAT3, phosphorylated at Tyr705, can translocate to the nucleus to regulate gene transcription, serving as a common risk factor in DN pathogenesis, promoting renal fibrosis, cellular senescence, and inflammation [45, 46]. Additionally, recent studies have shown that reducing the levels of pSTAT3/STAT3 can repress the senescence and apoptosis of renal tubular epithelial cells in DN [47]. A recent study by Lv et al. found that EZH2 increased phosphorylation and methylation of STAT3 in autosomal dominant polycystic kidney disease [48]. However, little is known regarding the relationship between EZH2 and STAT3 and their potential roles in podocyte injury and senescence in DN. Based on existing studies, we speculate that EZH2 may mediate podocyte injury and aging in DN by interacting with STAT3.

In our study, an elevated pSTAT3/STAT3 ratio was detected in the DN models, inhibition of EZH2 decreased phosphorylation of STAT3. In addition, direct binding between EZH2 and STAT3 proteins in podocytes was also detected in our study, and this binding can be disturbed by GSK126. We also determined that the STAT3 methylation was relevant with EZH2 expression. Genetically knockdown of EZH2 could decrease the methylation of STAT3 in HG-stimulated MPCs. Subsequently, we regulated abundance of EZH2 and STAT3 in our DN models, and the results showed that overexpression of STAT3 counteracted the renoprotective effect of inhibiting EZH2 production, suggesting that STAT3 is a crucial mediator of EZH2-mediated podocyte senescence and injury in DN, and potentially acts through a non-histone methylation pathway. Notably, the combined suppression of EZH2 and STAT3 yielded a more potent podocyte-protective effect. This also suggested that the regulation of STAT3 signaling is complicated and is influenced by diverse factors. However, the mechanism through which STAT3 methylation affects STAT3 phosphorylation remains unclear. Previous studies have evidenced EZH2 can dimethylate K49 and trimethylate K180 of STAT3, while the K140 of STAT3 is methylated by SET domain containing lysine methyltransferase 9 (SET9) and demethylated by lysine-specific demethylase 1 [16, 49, 50]. The amino acid sequence around the K49 methylation site of STAT3 is highly similar to that around the H3K27 EZH2-dependent methylation site [49]. It has been suggested that methylation of STAT3 enhances its nuclear retention, chromatin-binding, and trans-acting activities [17]. Tyr705 phosphorylation is required for dimethylation of STAT3 as reported in current study [49]. Interestingly, our results also found the binding and colocalization existed involving EZH2 and pSTAT3 in podocytes, which indicated to us that EZH2 reinforced persistent STAT3 activity probably through blocking nuclear pSTAT3 dephosphorylation via pSTAT3 methylation to some extent. This provided a new idea that EZH2 may continuously enhance the activity of STAT3 by inhibiting dephosphorylation of pSTAT3 through increasing its methylation. However, PRC2-independent EZH2 functions remain an emerging area.

At present, researches on EZH2 are predominantly focused on its oncogenic effects in a broad spectrum of cancers and several clinical trials of inhibitors targeted to EZH2 are undergoing. Recently, the renoprotective effect of EZH2 inhibitors (GSK126, 3-DZNeP, EPZ-6438, ZLD1039) in attenuating proteinuria, renal inflammation and fibrosis has been verified in multiple animal models, providing a foundation for clinical transformation [40, 51–54]. In this study, our results show that pharmacological inhibition of EZH2 with GSK126 protects against podocyte senescence and injury in experimental DN, suggesting the potential therapeutic role of small molecule inhibitor of EZH2 in DN. Current therapeutic options for DN primarily include renin-angiotensin system (RAS) inhibitors, sodium-glucose cotransporter 2 inhibitors, and aldosterone receptor antagonists. However, these interventions only slow the progression of kidney disease and cannot completely prevent the occurrence or advancement of DN [55]. Furthermore, the combinational therapy, such as the application of EZH2 inhibitors together with the RAS inhibitors, may enhance the treatment efficacy in DN.

Our study is not without limitations. First, although we demonstrate that EZH2 interacts with STAT3 and that high glucose increases the pan-methylation at lysine residues on STAT3 in an EZH2-dependent manner, we did not identify the specific lysine residue(s) of STAT3 that are methylated in podocytes under diabetic conditions. Mass spectrometry-based mapping and mutational analysis of candidate lysines were not feasible within the scope of the present study and will be important to definitively establish the molecular mechanism of STAT3 regulation by EZH2 in DN. Second, while our genetic and pharmacologic data support STAT3 as a critical downstream effector of EZH2 in podocytes, EZH2 has many additional chromatin and non-chromatin targets, and we cannot exclude contributions from other pathways to the observed phenotypes. Additionally, the application of catalytic-dead EZH2 in MPCs will support our viewpoint more comprehensively. Third, it was illustrated that the cumulative incidence of DN is higher in men (4.1%) compared with women (2.5%) and the progression in DN of males developed faster than females [56]. This is because beneficial effect from estrogen and adverse effect from androgen in the kidney. The specific metabolic requirements of male and female tissues may lead to different susceptibility to diseases. However, we only conducted experiments on male mice, while how EZH2 participates in the development of female DN requires more evidence.

In summary, our study illustrates, for the first time, that EZH2 mediates podocyte injury and senescence in DN at least in part through activating STAT3. EZH2 physically interacts with STAT3, enhances its phosphorylation, methylation and transcriptional activity. Pharmacological or genetic inhibition of EZH2 can reduce db/db proteinuria and delay DN progression, which is probably through reducing STAT3 activation. Therefore, targeted inhibition of EZH2 may be a potential strategy for treating DN. 

## Supplementary Information

Below is the link to the electronic supplementary material.

Supplementary Material 1 (DOCX 1.43 MB)

## Data Availability

All data supporting this study are included in the article and the supplementary materials.

## Abbreviations

DM: Diabetes mellitus
DN: Diabetic nephropathy
GBM: Glomerular basement membrane
EZH2: Enhancer of zeste homolog 2
PRC2: Polycomb repressive complex 2
H3K27me3: Histone H3 lysine 27 trimethylation
STAT3: Signal transducer and activator of transcription 3
SBE-β-CD: Sulfobutylether-β-Cyclodextrin
AAV: Adeno-associated virus vector
uACR: Urinary albumin/creatinine ratios
MPCs: Mouse podocyte cells
NG: Normal glucose
HM: High mannitol
HG: High glucose
KD: Knockdown
OE: Overexpression
qPCR: Quantitative real-time PCR
BSA: Bovine serum albumin
SA-β-gal: Senescence-associated β-galactosidase
TEM: Transmission electron microscopy
PAS: Periodic acid-Schiff
eGFR: Estimated glomerular filtration rate
Co-IP: Co-immunoprecipitation
HMT: Histone methyltransferase
RAS: Renin–angiotensin system
