# Supplementary material for: Histone methyltransferase EZH2 drives podocyte injury and senescence in diabetic nephropathy through STAT3 activation
Source: Cell Mol Life Sci. 2026 Mar 11;83(1):172. doi: 10.1007/s00018-026-06136-x (PMC13013736; doi:10.1007/s00018-026-06136-x)
Supplement: Supplementary file 1 — Supplementary Material 1 (DOCX 1.43 MB) [file 18_2026_6136_MOESM1_ESM.docx]

**Table S1. Brief information of antibodies used in western blotting**

| Antibodies | Product numbers | Manufacturers |
| --- | --- | --- |
| EZH2 | 5246 | CST, USA |
| STAT3 | 12640 | CST, USA |
| Phospho-STAT3 (Tyr^705^) | 9145 | CST, USA |
| H3 | ab201456 | Abcam, UK |
| Podocin | ab50339 | Abcam, UK |
| Podocin | ab181343 | Abcam, UK |
| Nephrin | ab216341 | Abcam, UK |
| B7-1 | ab238481 | Abcam, UK |
| Klotho | ab181373 | Abcam, UK |
| p21 | ab109199 | Abcam, UK |
| pan-methylated lysine | ab23366 | Abcam, UK |
| Synaptopodin | 21064-1-AP, | Proteintech, China |
| Desmin | 16520-1-AP | Proteintech, China |
| β-actin | 200068-8F10 | Zenbio, China |

**Table S2. Primer sequence of RT-qPCR**

| Gene | Species | Primer sequence (5′–3′) |
| --- | --- | --- |
| *Ezh2* | Mus musculus | F: ATGAAGCAGACAGAAGAGGAAA  R: GGATAGCCCTCTTAGCAAAGAT |


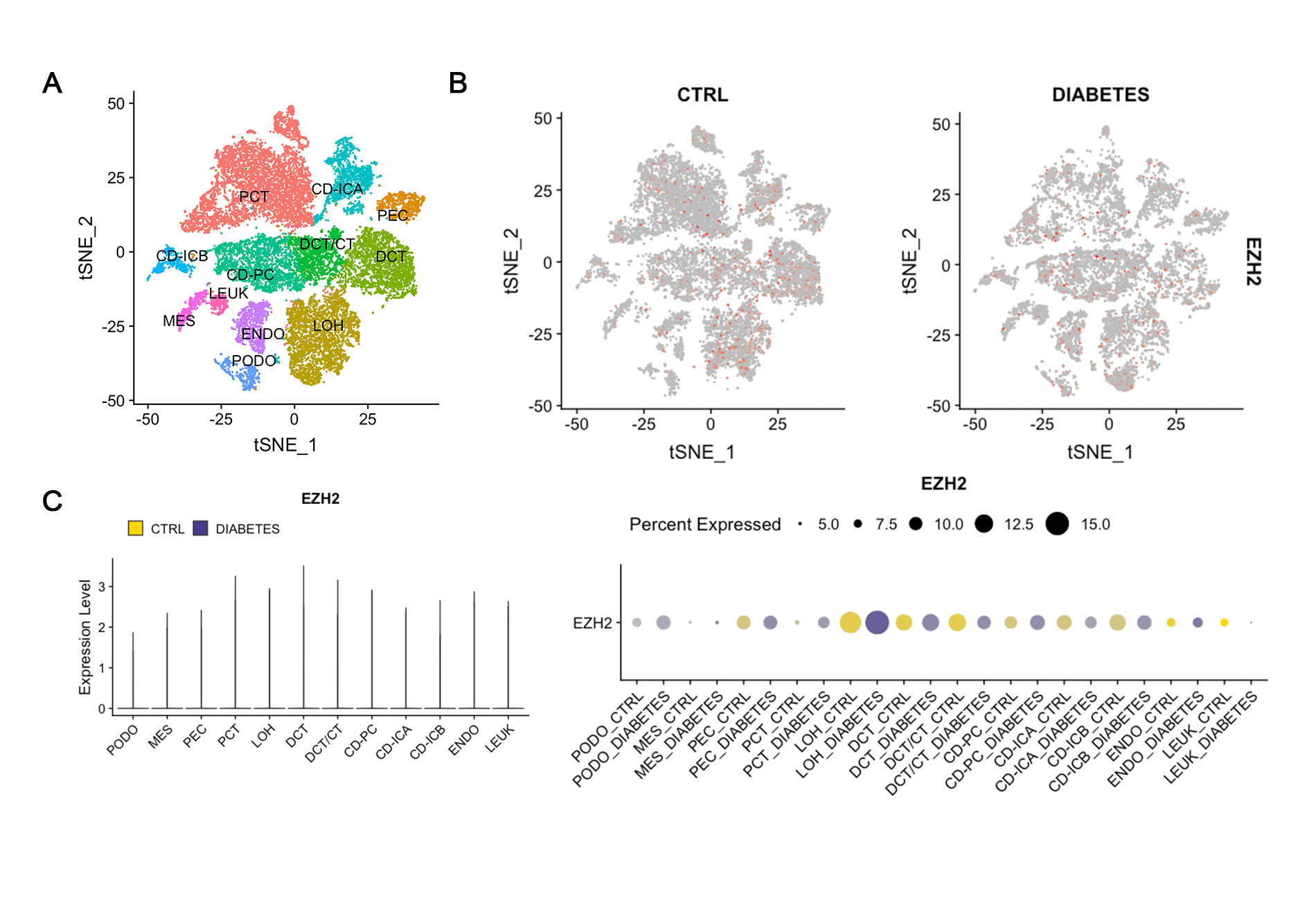


**Fig.S1 Expression of EZH2 in different types of renal cells of diabetic kidneys.** The single-cell RNA sequencing analysis of EZH2 based on the dataset Kidney Interactive Transcriptomics (<http://humphreyslab.com>). (A) tSNE plot showed the cluster of the integrated dataset of diabetic kidneys and control kidneys. PODO, podocyte; ENDO, endothelium; MES, mesangial cell; PCT, proximal convoluted tubule; LOH, loop of Henle; DCT, distal convoluted tubule; CFH, complement factor H; CT, connecting tubule; CD, collecting duct; PC, principal cell; IC, intercalated cell; LEUK, leukocyte. (B) Changes of EZH2 expression in each cell cluster in the control and DN groups. (C) Percentages of EZH2 levels in each cell cluster in the kidneys.

**
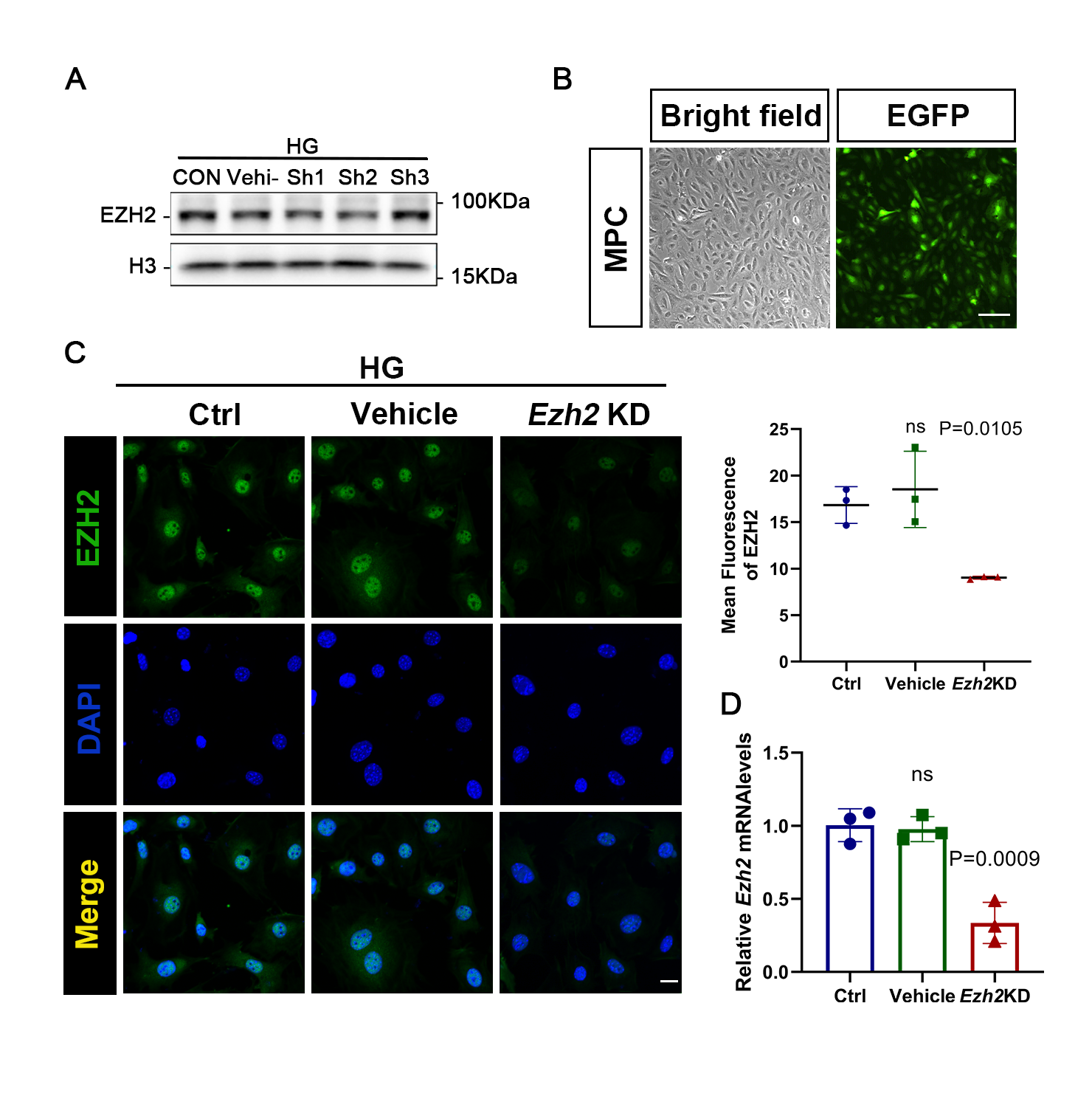
**

**Fig.S2 Effciency of EZH2 knockdown adenovirus in MPCs.** (A) Effect of three small hairpin RNAs (shRNAs) on inhibiting EZH2 in MPCs, as determined by western blotting. H3 served as the loading control. (B) Transfection efficiency of EZH2 shRNA2 adenovirus in MPCs. (C) Illustrative confocal microscopic images and quantification of EZH2 (green) in MPCs counterstained with DAPI (blue). Scale bar = 20 μm. ns: *P* > 0.05 vs. HG group. *P* values vs. HG group. (D) Relative mRNA levels of *Ezh2*, as determined by qPCR. ns: *P* > 0.05 vs. HG group. *P* values vs. HG group.
